# Isolation and Identification of E. cowanii from Powdered Infant Formula in NICU and Determination of Antimicrobial Susceptibility of Isolates

**Published:** 2014-06

**Authors:** Jalal Mardaneh, Mohammad-Mehdi Soltan-Dallal

**Affiliations:** 1 Prof Alborzi Clinical Microbiology Research Center, Namazee Hospital, Shiraz University of Medical Sciences, Shiraz; 2 Department of Bactriology, Department of Pathobiology and Microbiology, School of Public Health; 3 Food Microbiology Research Center, Tehran University of Medical Sciences, Tehran, Iran

**Keywords:** Powdered Infant Formula Milk; Neonatal Intensive Care Unit; E. Cowanii; Antimicrobial Susceptibility

## Abstract

***Objective:*** *Enterobacter cowanii* is a genus of common gram-negative, facultatively anaerobic, rod-shaped, non-spore-forming bacterium of the Enterobacteriaceae family. This organism can be potentially a powdered infant milk formula-borne opportunistic pathogen. The aim of this study was to isolate and identify *E. cowanii *from consumed powdered infant formula milk (PIF) in intensive care units (NICU) and to determine antimicrobial susceptibility patterns of this bacterium.

***Methods:*** *E. cowanii* was isolated according to FDA method in 125 samples of PIF milk purchased from drug stores between Jun 2011 and March 2012. For final confirmation, biochemical tests embedded in API-20E system were used. The drug susceptibility test was performed using the disc diffusion method according to CLSI recommendations.

***Findings*** ***:*** Out of the 125 PIF samples investigated, 4 (3.2%) samples were positive for *E. cowanii*. All four isolates from PIF samples were uniformly susceptible to imipenem, meropenem, ceftazidime, ciprofloxacin, and colistin. Fifty percent of isolates were resistant to ampicillin, amoxicillin, and cotrimoxazole

***Conclusion:*** Analysis of the results indicated that complementary studies are necessary to clarify the possible role of *E. cowanii *as a food contaminant, in common NICU infections and high risk groups including persons with underlying disease and immunocompromised individuals.

## Introduction

The genus Enterobacter in a family of Enterobacteriaceae was first described by Hormaeche and Edwards in 1960 and has undergone considerable taxonomic modification over the last 50 years^[^^1^^,^^2^^]^. The name *Enterobacter cowanii* is proposed for a group of microorganisms referred to as NIH group 42. The G + C content of its DNA ranges from 52.5% to 53.6%[3,4]. Because of distinct differentiation* of E. cowanii* by DNA hybridization methods from other members of Enterobacteriaceae family as well as its unique phenotypic and genotypic properties and since the DNA relatedness (5-38%) is closer to species of the genus *Enterobacter* than to other species of the Enterobacteriaceae, the members of NIH group 42 were placed in the genus *Enterobacter*^[^^3^^-^^5^^]^. The majority of *E. cowanii* strains were isolated from clinical and plant specimens^[^^3^^]^. 

*E. cowanii* is a genus of common gram-negative, facultatively anaerobic, rod-shaped, oxidase-negative, catalase positive, non-spore-forming bacterium of the Enterobacteriaceae family. It is motile by peritrichous ﬂagella[2,4]. This organism grows well on selective media for gram negative bacteria such as MacConkey agar and is facultatively anaerobic. *E. cowanii* is commonly found in ecological niches. This opportunistic pathogen is isolated from clinical specimens such as urine, sputum, blood, and pus. It was isolated by blood culture from a patient with gastric cancer. It may also be found in foods^[^^4^^,^^5^^]^. *E. cowanii* has been isolated from eucalyptus trees^[^^6^^,^^7^^]^.

 In recent years a remarkable increase in nosocomial infections has been reported especially in neonatal intensive care units, intensive care units and oncology departments. Underlying diseases, low-birth-weight, immunocompromised immune system, cancer chemotherapy, and intravenous catheterization can be predisposing factors in cases of infections due to unusual microorganisms, including *Enterobacter *spp. in neonates^[^^8^^-^^11^^]^*. *Infants admitted to NICUs, especially ones who have undergone surgery or have congenital abnormalities, are often at high risk for developing nosocomial infections. Clinical manifestations are often misleading and, in some circumstances, it may be difficult or even impossible to distinguish the source of the infection^[^^9^^,^^10^^]^. Enterobacteriaceae family members are potential PIF-borne pathogens. 

 Neonates and young children are exclusively vulnerable to infections caused by foodborne pathogens^[^^9^^,^^12^^]^. Contamination of PIF with *E. cowanii* will be associated with many diseases in neonates. Therefore, the microbiological safety of PIF is very important. Because PIF is not a sterile product, it is an excellent medium to support the bacterial growth. Bovine milk and plant materials are essential ingredients of PIF and a potential source of various bacteria that are pathogenic to neonates and adults^[^^12^^-^^14^^]^. The aim of our study was to isolate and identify antimicrobial susceptibility pattern of *E. cowanii *isolated from PIF in NICUs in Tehran hospitals.

## Subjects and Methods

**Place and Duration of Study:** Department of Pathobiology, School of Public Health, Tehran University of Medical Sciences, Tehran, between Jun 2011 and March 2012.

**Sampling:** A cross-sectional study was carried out on 125 samples of powdered infant formula milk (PIF) purchased from hospital drug stores in Tehran between Jun 2011 and March 2012. 

**Isolation and Identification:** PIF cans were surface sanitized with 70% ethanol and were opened in a laminar flow cabinet. Samples were taken from each product under aseptical conditions. *E. cowanii* was isolated according to FDA method[15,16]. We prepared 3 *Erlenmeyer *flasks of sterile distilled water (pre-warmed to 45ºC) at 9, 90 and 900 ml containing 1, 10 and 100 g of PIF, respectively. After the PIF was completely mixed and dissolved in distilled water, it was incubated at 37ºC for 18-24 h. Following incubation, 10 ml of each sample was added to 90 ml of Enterobacteriaceae enrichment (EE) broth medium and incubated at 37ºC for 18-24 h. After incubation, a lapful of the enrichment culture was streaked onto duplicates violet red bile glucose agar (VRBGA) plates and cultured at 37ºC for 18-24 h. A total of 4 suspicious colonies were picked from each VRBGA plate and pure culture was achieved. For detection of non-lactose fermenting isolates**, **presumptive colonies were streaked onto MacConkey agar and incubated at 37ºC for 72 h. For final confirmation biochemical tests were embedded in the API-20E biochemical kit system (Bio-Mérieux) and manual biochemical tests were used according to directions of the manufacturer**. **For long term storage, the purified isolates were stored in tryptic soy broth (TSB) with 20% glycerol (Merck Co.) at -20ºC. 

**Antibiotic sensitivity testing:** Antibiotic sensi-tivity testing was performed using Kirby-Bauer disk diffusion method on Mueller Hinton agar according to CLSI guidelines[17]. Antimicrobial agents used in this study are listed in Table 1. 

**Statistical analysis: **The calculation of sample size was performed by using McNemar's test. Data were analyzed using SPSS software, version 19.

***Findings***

Out of the 125 PIF investigated samples, 4 (3.2%) samples were positive for *Enterobacter cowanii.* The gram staining of the colony of organism showed gram negative rods. On VRBGA agar selective medium purple/pink colored colonies, and on MacConkey agar lactose fermenting, smooth, convex, punctuate, umbilicated, glistening colonies were grown during 16 to 20 hours. Isolated strains were oxidase negative, catalase positive, motile and produced other biochemical reactions which are characteristic of *E. cowanii* (Table 2). Fifty percent of isolates were resistant to ampicillin, amoxicillin, and cotrimoxazole. Susceptibility patterns of isolates are listed in Table 1.

## Discussion

*E. cowanii* shows the common characteristics of the genus *Enterobacter,* mostly isolated from different sources such as clinical specimens, foods, plants, in developed and developing countries worldwide[3-6]. One large difficulty in preventing NICU and hospital-acquired infections is to find the source of the infectious agent and its route of transmission. The consumption of powdered infant milk formula is wide-spreading; however, few studies have been undertaken to evaluate the role* of E. cowanii* in food safety. 

 To the best of our knowledge, there is no previous report on the isolation and identification *of E. cowanii *from PIF and determination of antimicrobial susceptibility pattern of this bacterium in Iran. In this study, we demonstrated that *E. cowanii* strains are widely spread in PIF. The results showed that all *E. cowanii* strains isolated from PIF samples were sensitive to meropenem, ceftazidime, ciprofloxacin, imipenem, chloram-phenicol, cefepime, levofloxacin, piperacillin, gentamicin, moxifloxacin, and colistin. In the present study 50% of isolates were resistant to ampicillin, amoxicillin, and cotrimoxazole**.**

**Table 1:** Antimicrobial susceptibility pattern of *Enterobacter cowanii* strains isolated from PIF (n=4)

**Antibiotic**	**Sensitive (%)**	**Intermediate (%)**	**Resistant (%)**
**Ampicillin (AP)**	1 (25)	1 (25)	2 (50)
**Amoxicillin (A)**	1 (25)	1 (25)	2 (50)
**Aztreonam (ATM)**	3 (75)	1 (25)	-
**Cefotaxime (CTX)**	1 (25)	2 (50)	1 (25)
**Amikacin (AK)**	3 (75)	1 (25)	-
**Streptomycin (S)**	3 (75)	1 (25)	-
**Meropenem (MEM)**	4 (100)	-	-
**Mezlocillin (MEZ)**	2 (50)	2 (50)	-
**Nalidixic acid (NA)**	3 (75)	1 (25)	-
**Tigecycline (TGC)**	3 (100)	1 (25)	-
**Tetracycline (T)**	3 (75)	1 (25)	-
**Ticarcillin (TC)**	3 (75)	1 (25)	-
**Chloramphenicol (C)**	4 (100)	-	-
**Ceftazidime (CAZ)**	4 (100)	-	-
**Ciprofloxacin (CIP)**	4 (100)	-	-
**Cefepime (CPM)**	4 (100)	-	-
**Imipenem (IMI)**	4 (100)	-	-
**Levofloxacin (LEV)**	4 (100)	-	-
**Minocycline (MN)**	3 (75)	1 (25)	-
**Piperacillin (PRL)**	4 (100)	-	-
**Piperacillin-tazobactam (PTZ)**	2 (50)	2 (50)	-
**Carbenicillin (PY)**	1 (25)	-	3 (75)
**Tobramycin (TN)**	3 (75)	1 (25)	-
**Cotrimoxazole (TS)**	2 (50)	-	2 (50)
**Moxifloxacin (MFX)**	4 (100)	-	-
**Gentamicin (GM)**	4 (100)	-	-
**Colistin (CO)**	4 (100)	-	-

**Table 2:** Biochemical reactions of *Enterobacter cowanii*

Test	Reaction/Result
Gram stain	Gram-negative, rod
Triple sugar iron agar	Acid (Yellow) slant/Acid (Yellow) butt. No H2S
Motility	**+**
Oxidase	**-**
Catalase	**+**
Nitrate reduction	**+**
Simmons citrate’s at 37°C	**+**
Gas from glucose	**+**
Acid from glucose	**+**
Lactose	**+**
Maltose	**+**
Sucrose	**+**
Sorbitol	**+**
Mannitol	**+**
Xylose	**+**
Raffinose	**+**
Arabinose	**-**
D-Cellobiose	**+**
Malonate	**-**
Adonitol	**-**
Rhamnose	**+**
a-Methyl-D-glucoside	**-**
Salicin	**+**
Inositol	**-**
Indole	**-**
Methyl red (MR)	**-**
Voges proskauer (VP)	**+**
Urease	**-**
Lysine decarboxylase	**-**
Ornithine decarboxylase	**-**
Arginine dehydrolase	**-**
Phenylalanine deaminase	**-**
Esculin hydrolysis	**+**
Gelatine hydrolysis	**-**
ONPG	**+**
DNAase	**-**

 To the best of our knowledge, there is no previous report on the **isolation and identification ***of E. cowanii ***from PIF and determination of antimicrobial susceptibility pattern of this bacterium in Iran**. In this study, we demonstrated that *E. cowanii* strains are widely spread in PIF. **The results showed that all ***E. cowanii*** strains isolated from PIF samples were sensitive to **meropenem, ceftazidime, ciprofloxacin, imipenem, chloram-phenicol, cefepime, levofloxacin, piperacillin, gentamicin, moxifloxacin, and colistin. In the present study 50% of isolates were resistant to ampicillin, amoxicillin, and cotrimoxazole**.**

*E. cowanii* is an opportunistic organism and, when introduced into the human organs or other fauna, may cause infection. Disease caused by this organism can occur in individuals with underlying diseases especially patients hospitalized in NICU^[^^10^^]^. Confirmed virulence of *E. cowanii *is difficult to reveal, because clinical reports involving *E. cowanii *are typically of polymicrobial nature, often involve patients that are already affected by diseases of other origin, lack pathogenicity confirmation, and diagnostic isolates are rarely conserved for confirmatory analysis. The results of the present study helps to better understanding of the role of *E. cowanii* as an opportunistic pathogen-causing disease in NICU. 

 Neonates and high risk groups (e.g. immunosuppressed and HIV positive individuals, senile persons) are particularly assailable to foodborne opportunistic pathogens^[^^18^^,^^19^^]^. Low birth weight infants in neonatal intensive care units are typically immunocompromised patients and their immunity is not fully mature. Therefore they are susceptible to different hospital-acquired infections^[^^10^^,^^20^^-^^22^^]^. In newborn infants, local natural barriers against bacterial infections are compromised and the production of secretory immunoglobin A is absent during the first days of life^[^^9^^]^. The decreased production and function of local and systemic defense depends on antigen exposure and contributes to greater susceptibility to bacterial infection during the neonatal period^[^^9^^,^^23^^]^. From our results we conclude that *E. cowanii *may be able to start a hospital outbreak, through powdered infant milk formula. The inherent capability of this organism to remain viable and grow well at room temperature may contribute to such contamination. Caregivers in hospital neonatal units should be constantly alerted to the fact that powdered infant formula products are not sterile and may be colonized with different microorganisms. 

 In addition, infant formula producers must accomplish guidelines aimed to decrease the risks of products contamination with foodborne pathogens. Controlling the primary populations of *E. cowanii* during the PIF production process and preventing post processing contamination by using suitable microbiological guidelines, is accessible. Sanitary practices for the preparation of infant formula in both the home and hospitals *should be carefully* controlled.

## Conclusion

Powdered infant milk formula containing members of the Enterobacteriaceae might impose additional risk of infection to the neonates and especially to the low birth weight premature babies. There is very little information about virulence factors and pathogenicity of *E. cowanii* in human, so complementary studies are necessary to clarify the possible role of *E. cowanii *as a food contaminant, in common NICU infections and high risk groups including persons with underlying disease and immunocompromised individuals.

## Authors’ Contribution:

All authors listed have contributed sufficiently to the project to be included as authors, and all those who are qualified to be authors are listed in the author byline. 
